# Statins and Fibrates for Diabetic Retinopathy: Protocol for a Systematic Review

**DOI:** 10.2196/resprot.6650

**Published:** 2017-02-22

**Authors:** Vania Mozetic, Carolina Gomes Freitas, Rachel Riera

**Affiliations:** ^1^ Universidade Federal de Sao Paulo Sao Paulo Brazil

**Keywords:** diabetic retinopathy, hypolipidemic agents, hydroxymethylglutaryl-CoA reductase inhibitors, fibric acids, ETDRS, HMG-CoA reductase inhibitors

## Abstract

**Background:**

Diabetic retinopathy (DR) is a common microvascular complication of diabetes mellitus, and more than 75% of patients who have had diabetes for more than 20 years will have some degree of DR. This disease is highly destructive to self-esteem and puts a high burden on public health and pension systems due to the effects that it has on people of working age. The current mainstay of treatment is laser photocoagulation, which causes impairment of vision and discomfort to patients. Thus, finding a systemic drug that could act on all microcirculation and prevent direct manipulation of the eyes would be highly desirable.

**Objective:**

To assess the efficacy and safety of the drugs in the statin and/or fibrate groups for the prevention and treatment of DR.

**Methods:**

In this systematic review, we will select randomized controlled trials of fibrates or statins used for the treatment or prevention of DR. Our search strategy will include free text terms and controlled vocabulary (eg, MeSH, Emtree) for, “diabetic retinopathy”, “statins”, “fibrates”, “hypolipidemic agents”, and for drugs from both groups. Databases that will be used include Medical Literature Analysis and Retrieval System/PubMed, Embase, Cochrane Central Register of Controlled Trials, Latin American and Caribbean Center on Health Sciences Information, Clinicaltrials.gov, World Health Organization International Clinical Trials Registry Platform, and OpenGrey, and we will not have language or date limits. Two review authors will independently select eligible studies and assess the risk of bias using the Cochrane Collaboration’s tool. We will report structured summaries of the included studies and, if possible, conduct meta-analyses.

**Results:**

This is a protocol for a systematic review, therefore results are not available. We registered a short version of this protocol before progressing in the review and we are currently in the process of selecting the studies for inclusion.

**Conclusions:**

Intensive glucose control and lowering blood pressure and lipids are mechanisms that protect macrocirculation in diabetic patients. Both macrovascular and microvascular events in diabetic patients appear to have a common pathway, starting with endothelial injury. Thus, prevention and treatment of microvascular events may benefit from the same interventions. In the review for which we have written this protocol, we will assess whether the use of lipid-lowering oral drugs of the statin and/or fibrate groups may prevent and/or retard progression of DR, with the added benefit of preserving visual acuity.

**Trial Registration:**

PROSPERO CRD42016029746

## Introduction

Diabetic retinopathy (DR) is a common microvascular complication of diabetes mellitus, and more than 75% of patients who have had diabetes for more than 20 years will have some degree of DR [1]. This disease is the leading cause of blindness among working-age Americans [2]. It is estimated that 33,000 new cases of diabetic macular edema, 86,000 new cases of proliferative DR, and 12,000-14,000 new cases of blindness are caused by DR each year in the United States alone [1]. Projections compute that by 2050 the number of Americans aged 40 years and older with DR and vision-threatening DR will triple compared to 2005 numbers (from 5.5 million to 16.0 million for the former and 1.2 million to 3.4 million for the latter) [3].

A putative pathophysiological mechanism of the disease is through products of nonenzymatic glycosylation, named advanced glycosylation end products (AGEs) [4,5]. AGEs are proteins or fats that become glycated after exposure to sugar, and the oxidative stress that occurs in diabetes is one of the probable causes that triggers this reaction [6]. AGEs bind to receptors in the retinal vessel endothelium and trigger the extrinsic pathway of coagulation, along with the inhibition of protein C (a physiological anticoagulant), and increase production of endothelin-1 (a potent vasoconstrictor) [7]. Together, these cascades lead to narrower vessels, increased permeability of the vascular wall, and consequently tissue ischemia. Ischemia, in turn, attracts angiogenic factors that promote neovascularization [8]. Lipids also presumably play an important role in the exudative stage of DR. The increased permeability of retinal capillaries causes extravasation of plasma lipoproteins that (along with degenerating cells) are engulfed by macrophages and form hard exudates, which is a defining characteristic of this stage of the disease [9].

Numerous studies on the newer antivascular endothelial growth factor drugs for the treatment of DR have been conducted, but laser photocoagulation persists as the treatment with the highest level of efficacy and safety [10]. However, even being the first option of treatment, laser photocoagulation is frequently associated with irreversible side effects caused by the ablation of retinal tissue. Visual field loss and impairment of night vision are frequent, and the procedure itself is very painful [11]. In this capacity, systemic drugs are highly desirable since they might prevent the onset and progression of DR, thereby avoiding the harms of manipulating the eye. Drugs might also allow for the chance to act in a preventive manner across the entire vascular endothelium.

It is known that all vascular diabetic alterations, whether in macrocirculation or microcirculation, have a common endothelial start. It is also well known that inadequate glycemic control, hypertension, and dyslipidemia are risk factors for the development of macrovascular disease in diabetics. Although the importance of the first two factors in microcirculation have already been addressed by previous systematic reviews [12,13], the relationship between lipids and the development and severity of DR is complex and remains unclear [14].

A recent update of The Wisconsin Epidemiologic Study of Diabetic Retinopathy, a 30-year follow-up of approximately 903 patients, found no association between total cholesterol or high density lipoprotein (HDL) and incidence of DR or macular edema, while there was a modest association between higher levels of HDL and decreased prevalence of proliferative DR [15]. The investigators then concluded that total cholesterol and HDL (as well as statin use) had a modest impact on DR [15]. Studies examining dietary lipid interventions showed favorable variable results in DR progression [16-18]. Other studies with clofibrate found reductions in hard exudates, but did not find differences in visual acuity [19-21].

Conversely, some randomized controlled trials (RCTs) showed beneficial effects of hypolipidemic drugs, suggesting that they may slow the progression of DR. The Action to Control Cardiovascular Risk in Diabetes (ACCORD) Eye Study, for example, evaluated the effects of specific strategies for managing blood glucose levels, serum lipid levels, and blood pressure on cardiovascular events in participants with type 2 diabetes [22]. This study also assessed the effects of these medical strategies on the progression of DR in a subgroup of trial participants: results showed that patients with type 2 diabetes who received fenofibrate and simvastatin had less progression of DR at 4 years when compared to placebo (6.5% vs 10.2% respectively; *P*=.006) [22]. Similarly, the Fenofibrate Intervention and Event Lowering in Diabetes study concluded that monotherapy with fenofibrate resulted in a significant reduction in the need for laser therapy at 5 years for either macular edema or proliferative retinopathy when compared to the placebo group (3.4% vs 4.9%; *P*<.001) [23]. Nevertheless, in the study that followed ACCORD patients (known as the Action to Control Cardiovascular Risk in Diabetes Follow-On Eye Study), this difference disappeared 8 years after randomization in the original ACCORD study (11.8% vs 10.2% respectively; *P*=.60) [22].

In light of these conflicting results, and considering the absence of the highest level of evidence for this question, it is critical to summarize the efficacy and safety of statins and fibrates for the prevention and treatment of DR through a systematic review of RCTs.

### Objectives

To assess the efficacy and safety of the drugs of the statins and/or fibrates groups for the prevention and treatment of DR.

## Methods

This protocol is registered in PROSPERO (CRD42016029746). We developed the protocol according to the Cochrane Handbook of Interventions Reviews [24] and report it according to the Preferred Reporting Items for Systematic Review and Meta-Analysis (PRISMA) Protocols [25].

### Types of Studies

For the purposes of this systematic review, only RCTs will be included. Given the progressive nature of the clinical situation, cross-over designs will not be considered.

### Types of Patients

Patients with type 1 or 2 diabetes, with or without nonproliferative retinopathy (for treatment and prevention, respectively) will be considered. Patients with proliferative retinopathy will be excluded.

### Types of Interventions

The interventions considered will be any drug from the statin or fibric acid groups, either in isolation or compared to placebo, no intervention, or a different type of statin or fibrate. We will also consider any statin or fibric acid as adjunctive therapy if we find RCTs of main therapy with statin or fibrate versus main therapy with placebo, no intervention, or a different type of statin or fibrate. Photocoagulation may be considered as a main therapy in this schema. In each situation (isolation or adjunctive) we will consider studies with any dose or any duration course of the intervention.

### Types of Outcome Measures

#### Primary Outcomes

Our primary outcomes will include: (1) aiming prevention, the proportion of patients that develop DR; (2) aiming treatment, the proportion of patients with progression of DR; and (3) aiming safety, the proportion of patients with at least one serious adverse event (ie, those that are immediately life-threatening, or resulted in hospitalization, incapacity, malignant disease, or death).

DR will be defined as 35 or more points in the Early Treatment Diabetic Retinopathy Study (ETDRS) scale [26], based on evaluation of stereoscopic color fundus photographs of the eyes of participants who did not have retinopathy at baseline. This score is equivalent to the categories of mild nonproliferative DR, or more severe DR [27].

Progression will be defined as a change from baseline of 2 or more steps on the same scale. We will accept trials that describe outcomes in terms of steps in the ETDRS scale and trials that described outcomes that can be converted to the ETDRS scale (eg, for older trials). For the adverse events outcome, we will not conduct additional searches in nonrandomized studies, which are not included in this review.

We plan to assess these outcomes at 2, 6, 12, 18, and 24 months, and annually thereafter, grouping the trials that fall within these time points (eg, group trials that assess the outcomes up to 2 months).

#### Secondary Outcomes

We will assess the proportion of patients with: (1) decrease of visual acuity (any decrease) measured by Snellen or LogMAR charts, and (2) proliferative DR (measured by the ETDRS scale) with at least one minor adverse event (ie, adverse events not included in the serious adverse event outcome). We will also evaluate quality of life measured by the National Eye Institute Visual Functioning Questionnaire 25 or another validated vision-related scale. These outcomes will be assessed at the same time points as the primary outcomes.

### Methods for Search

#### Electronic Search

We will systematically search the following databases: Medical Literature Analysis and Retrieval System (via PubMed), Embase (via Elsevier), Latin American and Caribbean Center on Health Sciences Information (via Virtual Health Library), and Cochrane Central Register of Controlled Trials (via Wiley). The search strategy will include controlled vocabulary (eg, MeSH, Emtree) and free-text terms related to, “diabetic retinopathy”, “hypolipidemic agents”, “statins”, “fibrates”, and drugs from both groups. No limits for data, language, or status of the publication (eg, conference abstracts, full-text, ongoing studies) will be used. Additional searches will be conducted in the clinical trial registries of Clinicaltrials.gov and the World Health Organization International Clinical Trials Registry Platform, and in the grey literature source OpenGrey.

#### Hand Search

We will assess reference lists of all included studies and review articles for additional references. We will contact authors of identified trials and ask them about other published and unpublished studies. We will also contact manufacturers and specialists in the field of ophthalmology.

### Selection of Studies

Two authors (VM and CGF) will independently read the references and select the studies according to the inclusion and exclusion criteria. We will exclude duplicates and collate multiple reports of the same study so that each study, rather than each report, will be the unit of interest in the review. After the initial step of screening titles and abstracts of the records, we will read the full text articles for the potentially includible studies, finally deciding on the included ones and giving reasons for the exclusions in this step. A third reviewer (RR) will resolve any disagreements. We will record the selection process in sufficient detail to fulfill a PRISMA flow diagram and a *characteristics of excluded studies* table [28].

### Data Extraction and Management

We will use a standard data collection form for extracting study characteristics and outcome data. Two reviewers (VM and CGF) will extract the study characteristics outlined in Textbox 1.

**Characteristics that will be extracted from the included studies.**

- Methods: study design, total duration of study and run-in, number of study centers and location, study setting, withdrawals, and date of study.
- Participants: N, mean age, age range, gender, severity of condition, diagnostic criteria, inclusion criteria, and exclusion criteria.
- Interventions: intervention, comparison, concomitant medications, and excluded medications.
- Outcomes: primary and secondary outcomes (the final outcomes reported and those planned), and time points reported.
- Notes: funding for trial and notable conflicts of interest of trial authors.

One reviewer (VM) will copy the data from the data collection form into the Review Manager (RevMan 5.3) file [28]. We will double check that the data is entered correctly by comparing the study reports with how the data is presented in the systematic review.

### Assessment of Risk of Bias in Included Studies

Two reviewers (VM and CGF) will independently judge the risk of bias of each study using the Cochrane Collaboration's tool for assessing risk of bias [29]. The tool comprises the following domains: (1) random sequence generation, (2) allocation concealment, (3) blinding of participants and personnel, (4) blinding of outcome assessment, (5) incomplete outcome data, (6) selective outcome reporting, and (7) other bias. Each domain will be judged as *high risk*, *low risk*, or *unclear risk* of bias according to the criteria described in the risk of bias table in the Cochrane Handbook [29]. We will consider blinding separately for different key outcomes when necessary (eg, regarding unblinded outcome assessment, risk of bias for all-cause mortality may be very different than that of a patient-reported quality of life scale [28]). When considering treatment effects, we will take into account the risk of bias of the studies that contributed to that outcome.

### Data Synthesis

We will use a software Review Manager (RevMan 5.3) to perform meta-analyses whenever possible. We plan to use random-effects meta-analyses except when involving up to 3 studies in the pooled estimate; a situation in which we will use fixed-effects. This approach is a change from our previous protocol, in which we would use fixed-effects models for homogeneous studies and random-effects otherwise; however, random-effects models result in pooled estimates similar to fixed-effects models when there is little or no heterogeneity within studies. Additionally, random-effects analyses provide poor estimates for the confidence interval when examining few studies or when the studies are small [30]. If we are not able to analyze studies due to a lack of data for any comparison, or high heterogeneity as specified in the *assessment of heterogeneity* section, we will report the result of each individual trial narratively.

### Measures of Treatment Effect

We will analyze dichotomous data as risk ratios and continuous data as mean differences or standardized mean differences. We will undertake meta-analyses only when meaningful (ie, if the treatment participants and the underlying clinical question are similar enough for pooling to make sense). If multiple trial arms are reported in a single trial, we will include only the relevant arms, and if two comparisons from the same trial (eg, *drug X vs placebo* and *drug Y vs placebo*) must be included in the same meta-analysis, we will halve the control group to avoid double counting [28].

### Dealing with Missing Data

We will contact authors or study sponsors to verify key study characteristics or to obtain missing numerical outcome data when possible (eg, when a study is identified as *abstract only).* If outcome data are missing in both intervention groups, but reasons for these are both reported and balanced across groups, important bias would not be expected unless the reasons have different implications in the compared groups. In dichotomous studies, the potential impact of missing data depends on the frequency or risk of outcomes. In continuous outcomes, the potential impact increases with the proportion of participants with missing data [29].

### Assessment of Heterogeneity

We will assess studies regarding clinical and methodological heterogeneity. If studies are deemed homogeneous in these criteria, we will conduct meta-analyses and analyze statistical heterogeneity by visual inspection of the forest plots, and use Chi-squared and I^2^tests. Results of Chi-squared <0.10 and I^2^>50% will be considered heterogeneous; in these cases, we will try to explain heterogeneity by the prespecified groups for the subgroup analysis and also by the possible findings of the assessment of publication bias [30].

### Assessment of Reporting Bias

If 10 or more studies are included in the meta-analysis, we will assess reporting biases using funnel plots and visually inspect the plots for asymmetry [29].

### Subgroup Analyses and Investigation of Heterogeneity

Subgroup analyses for the primary outcomes will be conducted and consider the following groups: the different types of diabetes, the different kinds of hypolipemic drugs (statins, fibrates), the different doses of these drugs (eg, high-dose fibrates), and the dyslipidemia and/or diabetic macular edema status of the patients [30].

### Sensitivity Analyses

Sensitivity analyses will be conducted to determine the impact of exclusion of studies with overall high risk of bias. Such studies will include those judged to harbor *high risk* of bias in at least one of the main domains in the Risk of Bias Table (generation of randomization sequence, allocation concealment, and blinding) [30].

### Summary of Findings

Using GRADEpro software we will generate two summary of findings (SoF) tables, one for each key question of this review: development and progression of DR. We will use the five Grading of Recommendations Assessment, Development, and Evaluation criteria (study limitations, consistency of effect, imprecision, indirectness, and publication bias) to assess the quality of the body of evidence that contributes data to the meta-analyses for the prespecified outcomes. We will use the methods and recommendations described in Section 8.5 [29] and Chapter 12 [31] of the Cochrane Handbook for the judgment of these criteria. We will justify all decisions to downgrade or upgrade the quality of studies using footnotes, and make comments to aid readers’ understanding of the review when necessary. We will consider whether there is any additional outcome information that was not incorporated into meta-analyses, note this in the comments, and state if it supports or contradicts the information from the meta-analyses. The SoF table regarding the prevention of DR will comprise the following outcomes: (1) the proportion of patients with DR, (2) the proportion of patients with at least one serious adverse event, (3) the proportion of patients with proliferative DR, and (4) the proportion of patients with any decrease in visual acuity. The comparison for this table will be hypolipidemic drugs versus placebo or no intervention.

The SoF table regarding the treatment of DR will comprise the following outcomes: (1) the proportion of patients with progression of DR, (2) the proportion of patients with at least one serious adverse event, (3) the proportion of patients with proliferative DR, and (4) the proportion of patients with any decrease in visual acuity. The comparison for this table will be laser photocoagulation with hypolipidemic drugs versus laser photocoagulation with placebo or no intervention. The outcomes for both SoF tables will be reported at 6 months and 5 years (short- and long-term, respectively).

## Results

This is a protocol for a systematic review, therefore results are not available. We registered a short version of this protocol before progressing in the review and we are currently in the process of selecting the studies for inclusion.

## Discussion

Dyslipidemia is one well-known risk factor for the development of vascular disease in diabetics. However, to date the effects of statin and/or fibrate use have not been addressed by a systematic review. The findings of this review will provide an assessment of the existing evidence for patients and health care providers that deal with this severe and prevalent complication of diabetes.

## Abbreviations

ACCORD: Action to Control Cardiovascular Risk in Diabetes
AGE: advanced glycosylation end products
DR: diabetic retinopathy
ETDRS: Early Treatment Diabetic Retinopathy Study
HDL: high density lipoprotein
PRISMA: Preferred Reporting Items for Systematic Review and Meta-Analysis Protocols
RCT: randomized controlled trial
SoF: summary of findings
